# Botulinum Toxin A and Lower Urinary Tract Dysfunction: Pathophysiology and Mechanisms of Action

**DOI:** 10.3390/toxins8040120

**Published:** 2016-04-21

**Authors:** Jia-Fong Jhang, Hann-Chorng Kuo

**Affiliations:** Department of Urology, Buddhist Tzu Chi General Hospital, Tzu Chi University, 707 Chung-Yang Road, Section 3, Hualien 970, Taiwan; alur1984@tzuchi.com.tw

**Keywords:** interstitial cystitis, overactive bladder, treatment

## Abstract

The use of onabotulinumtoxinA (BoNT-A) for the treatment of lower urinary tract diseases (LUTD) has increased markedly in recent years. The indications for BoNT-A treatment of LUTD now include neurogenic or idiopathic detrusor overactivity, interstitial cystitis/bladder pain syndrome and voiding dysfunction. The mechanisms of BoNT-A action on LUTDs affect many different aspects. Traditionally, the effects of BoNT-A were believed to be attributable to inhibition of acetylcholine release from the presynaptic efferent nerves at the neuromuscular junctions in the detrusor or urethral sphincter. BoNT-A injection in the bladder also regulated sensory nerve function by blocking neurotransmitter release and reducing receptor expression in the urothelium. In addition, recent studies revealed an anti-inflammatory effect for BoNT-A. Substance P and nerve growth factor in the urine and bladder tissue decreased after BoNT-A injection. Mast cell activation in the bladder also decreased. BoNT-A-induced improvement of urothelium function plays an important mitigating role in bladder dysfunction. Vascular endothelial growth factor expression in urothelium decreased after BoNT-A injection, as did apoptosis. Studies also revealed increased apoptosis in the prostate after BoNT-A injection. Although BoNT-A injection has been widely used to treat different LUTDs refractory to conventional treatment, currently, onabotulinumtoxinA has been proven effective only on urinary incontinence due to IDO and NDO in several large-scale clinical trials. The effects of onabotulinumtoxinA on other LUTDs such as interstitial cystitis, benign prostatic hyperplasia, dysfunctional voiding or detrusor sphincter dyssynergia have not been well demonstrated.

## 1. Introduction

Normal lower urinary tract function promotes efficient voluntary urine emptying and low-pressure urine storage, which contribute to continence [1]. The symptoms of dysuria, urinary frequency, and incontinence are signs of lower urinary tract diseases (LUTD) [2]. LUTD is a common health problem and its prevalence increases with age. A recent cross-sectional study revealed 61.2% of men and 61.1% of women reported LUTD [3]. The pathophysiology of LUTD is usually complicated, and the same symptom in different patients may have a different pathogenesis. Conventionally, LUTD is classified as originating from the bladder or bladder outlet. Oral medications such as alpha-adrenergic blockers and anti-muscarinic agents are effective first-line treatments for LUTD in many patients. However, some patients with LUTD are refractory to these conventional drugs.

Botulinum toxin (BoNT) is a potent neurotoxin produced by the bacterium *Clostridium botulinum*. It inhibits the release of the neurotransmitter acetylcholine from nerve fibers, thereby inhibiting muscle contractions. Because of these toxic effects, BoNT is widely used in medical practice [4]. Therapeutic onabotulinumtoxinA was first injected to correct strabismus in 1983 [5]. Currently, it is broadly applied to treat many neuromuscular and neuropathic pain diseases that are difficult to treat with conventional drugs [6,7]. The first application of onabotulinumtoxinA for the treatment of LUTD was in patients with spinal cord injury (SCI) and detrusor sphincter dyssynergia (DSD). Nowadays, onabotulinumtoxinA is used to treat urinary incontinence due to detrusor overactivity (DO). OnabotulinumtoxinA injection into the bladder wall (detrusor muscle) has been a breakthrough in treating refractory DO. The United States Food and Drug Administration approved this indication in 2013 [8,9]. The advantages of onabotulinumtoxinA injection are not only minimal invasiveness, but also provide an opportunity for the patients with LUTD in which conventional treatment has failed. Even with such widespread use, the mechanisms of onabotulinumtoxinA action in treating LUTDs remain unclear and involve many different pathways. Thus, the aim of this review is to introduce the pathophysiology of LUTD and describe the mechanisms of onabotulinumtoxinA effects in the treatment of LUTD.

## 2. Mechanism and Biology of OnabotulinumtoxinA

BoNT is the most potent biological toxin, and it was first isolated more than 100 years ago [10]. Seven different strains of *Clostridium botulinum* produce seven immunologically distinct neurotoxins (types A to G) [11], and the most common medically used type is BoNT-A. BoNT-A is a protein consisting of a 50-kDa light chain and a 100-kDa heavy chain connected by a disulfide bond [12]. In the presynaptic nerve membrane, the *C*-terminal of the heavy chain of BoNT-A binds to synaptic vesicle protein 2 (SV2), and then BoNT-A is taken into the nerve terminal by endocytosis [13]. In the endosomal vesicle, the light chain and heavy chain separate. The *N*-terminal of the heavy chain binds to the endosomal membrane where it induces the light chain to be transported from the endosome to the cytosol [14]. The light chain is the biologically active moiety of BoNT-A.

In the presynaptic nerve terminal, neurotransmitters are released by exocytosis and mediated by soluble *N*-ethylmaleimide-sensitive factor attachment receptor protein complexes that consist of synaptobrevin, syntaxin and synaptosome-associated protein 25 (SNAP-25) [15]. The light chain of BoNT-A cleaves SNAP-25, inhibiting the release of the neurotransmitter acetylcholine by disrupting the fusion of vesicles with the neuron cell membrane, finally causing the flaccid paralysis of muscles [16]. In addition, studies also revealed BoNT-A blocks noxious neurotransmitter release, including substance P, calcitonin gene-related peptide and adenosine triphosphate (ATP) [17,18]. Recently, many clinical studies have proven the efficacy of BoNT-A in treating different neuropathic pain disorders, such as post herpetic neuralgia, trigeminal neuralgia and post-traumatic neuralgia [19].

BoNT-A injection has been widely used to treat different LUTDs refractory to conventional treatment. Currently, onabotulinumtoxinA has been proven effective only on urinary incontinence due to IDO and NDO in several large-scale clinical trials. The effects of onabotulinumtoxinA on other LUTDs such as IC/BPS, BPH, DV, DSD have not been well demonstrated.

## 3. Mechanism Action of BoNT-A on Bladder Disease

Symptoms only are not a reliable tool for diagnosis of LUTD. Symptoms, such as a weak urine stream, could be the result of diseases originating from the bladder or bladder outlet. Further examinations, such as uroflowmetry or bladder sonography, are usually necessary to confirm the diagnosis [20]. LUTDs originating from the bladder include DO, interstitial cystitis/bladder pain syndrome (IC/BPS) and hypersensitive bladder. Even today, the details of the complete pathogenesis of many LUTDs remain an enigma, and this is why these diseases are sometimes difficult to treat successfully.

### 3.1. Pathophysiology and Mechanism of BoNT-A in DO

The International Continence Society defines DO as an urodynamic observation characterized by involuntary detrusor contractions during the bladder-filling phase that may be spontaneous or provoked [2]. Patients with DO usually suffer from symptoms of urgency, which is defined as a complaint of sudden, compelling desire to pass urine that is difficult to defer [21]. Disturbances in or injury to nerves, the detrusor muscle or urothelium can cause DO [22]. Clinically, the cause is classified as neurogenic (NDO) in patients with the relevant neurological conditions or idiopathic (IDO) in patients without definitive cause [21]. Bladder contraction is mediated by the parasympathetic nervous system via the release of predominantly acetylcholine, which interacts with muscarinic receptors on the detrusor muscle [22]. Parasympathetic nerves also release adenosine triphosphate (ATP) and stimulate the purinergic receptors (P2X) in the detrusor to induce contraction [23].

In central nervous system (CNS), detrusor contraction may be inhibited by the prefrontal cerebral cortex, the L-region of the pontine micturition center and the lumbar spinal cord [22]. These inhibitory neurons in the CNS release gamma-aminobutyric acid and glycine, inhibiting detrusor contraction [24]. A lesion above the lumbosacral cord level interrupts voluntary control of micturition and results in NDO due to dysfunction of the inhibitory pathway. In addition, a sacral spinal reflex, which is mediated by formerly silent capsaicin-sensitive unmyelinated C-fibers is also active in patients with SCI [24]. This reflex also plays a significant role in NDO. On the other hand, the pathogenesis of IDO is more complicated. Although the patients with IDO do not have any specific disease of the central or peripheral nervous systems, recent studies revealed neurologic dysfunction involving afferent pathways that contributed to the pathogenesis of IDO [24]. The bladder afferent pathways consist of mechanosensitive myelinated A fibers and chemosensitive unmyelinated C fibers [25]. Urothelial dysfunction could increase the afferent stimulation produced by bladder fullness and contribute to the perception of urgency, thereby contributing to activation of the abnormal detrusor contractions [26]. Increased expression of urothelium receptors such as P2X2, transient receptor potential cation channel subfamily V member 1 (TRPV1) and muscarinic receptors is associated with IDO in the human bladder [27].

Since Schurch first used intravesical BoNT-A injection in patients with SCI and NDO in 2000, many studies had proven its efficacy in reducing urgency and urgency incontinence [28]. In the efferent nerves of the bladder, BoNT-A injection blocks the release of acetylcholine from both preganglionic and postganglionic parasympathetic nerves by cleaving SNAP-25, thereby temporarily inhibiting detrusor muscle contraction [29]. Studies also showed that vesicular noradrenaline release was inhibited after BoNT-A injection in the bladder wall [30]. BoNT-A not only blocked neurotransmitter release after injection into the detrusor muscle but also reduced muscarinic receptor M2 in the nerve terminal [31]. An animal study also revealed BoNT-A block ATP release from purinergic efferent nerves in the detrusor muscle, which may have contributed to the effectiveness of BoNT-A in treating DO [32].

In addition to efferent nerve signals, increased afferent nerve signals are believed to be central to the pathophysiology of IDO and the generation of urgency [27]. When the urothelium is stretching, both acetylcholine and ATP are released from the basal lateral surface of the urothelium. This activates afferent nerves in the suburothelial layer and induces the micturition reflex [33]. Recently, increasing evidence suggests that BoNT-A blocks afferent signaling in the bladder [34]. In a study of the bladders of patients with SCI, onabotulinumtoxinA injection markedly reduced urothelial ATP release by 53% [35]. Valerie *et al.* also reported that onabotulinumtoxinA injection attenuated ATP release in the bladder with physiological and supra-physiological distension pressures [36]. Nitric oxide (NO) also inhibits afferent nerve conduction in the bladder detrusor, with increased NO release from the urothelium after onabotulinumtoxinA injection [36]. However, in the study by Valerie *et al.*, acetylcholine release from urothelium was not significantly decreased [36].

Evidence also shows that afferent nerve receptors in the suburothelium are regulated after onabotulinumtoxinA injection into the urothelium. A study of patients with DO revealed decreased expression of P2X3 in the bladder mucosa within four weeks after onabotulinumtoxinA injection [37]. Additionally, the decline in P2X3 receptors was significantly correlated with the reduction of urgency episodes four and 16 weeks after onabotulinumtoxinA injection. In another single-blind study of children with NDO, the purinergic receptors P2X2 and P2X3 in urothelium reached significant reductions (*p* < 0.05) after onabotulinumtoxinA injection [38]. Increased urothelial TRPV1 in patients with NDO may also play a role in the pathophysiology [39]. After onabotulinumtoxinA injection, a significant reduction of TRPV-1 in the suburothelial nerve fibers was reported [37]. In summary, the effect of BoNT-A in patients with DO involves efferent and afferent nerve pathways. Table 1 summarizes the primary mechanisms of action of BoNT-A on neurotransmitters (acetylcholine, ATP and NO) and receptors (M2, M3, P2X2, P2X3, and TRPV-1).

### 3.2. Pathophysiology and Mechanism of BoNT-A on IC/BPS

The clinical characteristics of IC/BPS are severe bladder pain associated with urgency, frequency, nocturia, dysuria and sterile urine [40]. Although many studies have focused on the pathophysiology of IC/BPS, it is still is a mystery in urology. Previously infection, autoimmune, central sensitization and urine abnormalities have been considered as etiologies of IC/BPS [41]. Recently, urothelial dysfunction and neurogenic inflammation have attracted great interest. A urothelial defect of surface glycosaminoglycan and associated changes in epithelial permeability are associated with the pathogenesis of IC/BPS [42,43]. Furthermore, as with DO, bladders affected with IC/BPS also had up-regulation of P2X3 receptors and increasing ATP release in the urothelium [44,45]. TRPV1 is the receptor of capsaicin stimulation and detects pain in the bladder [46]. The expression of TRPV1 in humans with IC/BPS is up-regulated and could be involved in the pathogenesis of IC/BPS [47]. The cell tight junction protein zonula occludens-1 (ZO-1) and adhesive junction protein E-cadherin are significantly decreased in the bladders of patients with IC/BPS but not in patients with DO [48]. Many studies revealed an increase in mast cell infiltration in the bladders of IC/BPS patients, which could play a central role in the pathogenesis of IC/BPS [49]. Substance P is a neurotransmitter secreted from the sensory nerve endings and is associated with inflammatory processes and pain [50]. Pang *et al.* reported significantly increased numbers of substance P-positive nerve fibers in the bladder submucosa of patients with IC/BPS [51]. Neurokinin-1 receptors, the high-affinity binding sites of substance P, were also upregulated in the bladders of patients with IC/BPS [52]. Substance P and mast cells play a pivotal role in the neurogenic inflammation of IC/BPS and induce inflammation in the bladder by the release of histamine and tumor necrosis factor alpha [51,53]. Nerve growth factor (NGF) is a neuropeptide involved in the regulation of growth; it is released by mast cells in the inflammation process NGF induces axonal outgrowth in pain neurons and leads to increased pain perception [54]. Increased NGF is found in the bladder mucosa, urine, and serum of patients with IC/BPS and is likely involved in the pathogenesis of IC/BPS [55,56].

OnabotulinumtoxinA injections in the bladder urothelium have been used in treating IC/BPS since 2004 [57]. A recent randomized, double-blind, placebo-controlled clinical trial revealed that intravesical injections of 100 U of onabotulinumtoxinA effectively reduced bladder pain symptoms in patients with IC/BPS [58]. Currently, the American Urology Association recommends intradetrusor onabotulinumtoxinA injections for treatment of IC/BPS in patients for whom conventional treatments failed [59]. BoNT-A injections improve urothelial function in patients with IC/BPS by decreasing P2X3 and TRPV1 receptor expression in the urothelium, which is a likely mechanism reducing pain in patients with IC/BPS [60]. Additionally, BoNT-A inhibits sensory neurotransmitter release further reducing pain sensation. CGRP is a potent peptide vasodilator that functions in the transmission of pain [61]. A study in rats revealed that BoNT-A injection into the bladder inhibited the evoked release of CGRP from afferent nerve terminals in the bladder, reducing pain [62]. In another study of acute and chronic rat bladder inflammation, BoNT-A injection also significantly inhibited the release of substance P and CGRP [63]. OnabotulinumtoxinA decreased NGF mRNA expression in the bladder mucosa of IC/BPS patients who responded to intravesical onabotulinumtoxinA injection [64]. The level of urinary NGF also decreased in these patients [65]. Pinto *et al.* also reported decreased urinary NGF after onabotulinumtoxinA injection into the trigone, even in patients with ulcerative IC/BPS [66]. Recent evidence also suggests onabotulinumtoxinA injections reduce bladder inflammation. Immunohistochemical studies reveal decreased tryptase expression in the urothelium after repeated injections, which suggests a reduction in active mast cells in the bladders of IC/BPS patients [67]. OnabotulinumtoxinA decreases the expression of vascular endothelial growth factor in the bladders of patients with IC/BPS and indicates attenuation of vasculogenesis in these patients [68]. Urothelial apoptosis is also declined after onabotulinumtoxinA injection. The expression of apoptosis regulation protein bcl-2-like protein 4 in the urothelium decreased within six months after onabotulinumtoxinA injection [68]. Additionally, the evidence of apoptotic signaling cascades also decreased [67,68]. Moreover, recent studies revealed peripheral BoNT-A injection has antinociceptive activity via CNS regulation [69] BoNT-A reaches the CNS by retrograde transport and blocks CNS synaptic transmission of glutamate, dopamine, ATP and gamma-aminobutyric acid [69]. Table 2 provides a summary of mechanisms of BoNT-A action in IC/BPS.

## 4. Mechanism Action of BoNT-A on Bladder Outlet Dysfunction

The main symptoms of bladder outlet obstruction are incomplete bladder emptying, diminished urinary stream, and post voiding urinary dribbling, which manifest in DSD, bladder neck dysfunction and benign prostatic obstruction (BPO) [70]. The anatomic structures of the bladder outlet include the bladder neck, urethral sphincter and prostate in men. Both the anatomic or functional obstruction of these structures results in slow urinary flow and an increase in intravesical pressure [70]. Anatomic disorders of bladder outlet obstruction such as BPO may be managed well with medical treatment or surgical intervention, such as resection of the prostate. However, the functional problems including DSD in patients with SCI, dysfunctional voiding (DV) or poor relaxation of the external urethral sphincter can be troublesome when conventional treatments fail [70].

DSD is characterized by involuntary contractions of the external urethral sphincter during a detrusor contraction caused by CNS injury between the pontine micturition center and the sacral spinal cord [71]. Transperineal onabotulinumtoxinA injection under electromyographic control was first used to treat such patients with SCI and DSD in 1997 [72]. Most urologists now use a rigid cystoscope with an injection needle to inject onabotulinumtoxinA directly into the urethral sphincter [73]. Although a large, randomized controlled trial is lacking, several preliminary studies suggest onabotulinumtoxinA injection for treatment of DSD due to SCI could result in higher voided urine volumes and lower voiding detrusor pressures [73]. In an earlier study of SCI with DSD, the amplitude of bulbosphincteric reflexes were decreased after urethral sphincter onabotulinumtoxinA injection and the latency also became normal [74]. An electromyographic study revealed static and dynamic urethral pressure in the patients with severe spasticity of the urethral sphincter decreased significantly after onabotulinumtoxinA injection [75]. When the bladder outlet resistance stemming from the urethral sphincter decreased, the voiding efficiency in the patients with DSD improved. The effect of BoNT-A on DSD is thought to be attributable to blocking acetylcholine release from presynaptic vesicles at the neuromuscular junction. This temporary, reversible chemo-denervation by BoNT-A relaxes the spastic external urethral sphincter in SCI patients with DSD [76]. Laboratory evidence confirming this mechanism of action of BoNT-A is needed. 

DV is characterized by an intermittent or fluctuating urine flow rate due to intermittent, involuntary contractions of the periurethral striated muscle during voiding in neurologically normal individuals [2]. Traditionally, DV has been considered a learned chronic disorder of childhood, presumably because the misguided habit of contracting the external urethral sphincter rather than relaxing it during voiding [77]. However, many adult patients with DV completely deny the existence of any voiding symptoms during their childhood [78]. Fowler *et al.* reported DV patients presenting with urinary retention, and the urethral sphincter electromyography of these patients demonstrated unexpected decelerating bursts and complex repetitive discharges [79]. On the other hand, some patients without such strong electromyographic activity might also have similar symptoms of DV due to poor relaxation of the pelvic floor muscle and urethral sphincter [80]. Relaxation of the bladder neck also is an important step of micturition. Bladder neck dysfunction leads to a weak urine stream and increases residual urine volume [80]. In the past, treating these voiding problems was difficult. However, recent studies suggest onabotulinumtoxinA injections in the external urethral sphincter, bladder neck or pelvic floor muscle offer promising prospects for improving DV symptoms [81,82,83]. OnabotulinumtoxinA injection into the rat urethral sphincter significantly inhibited acetylcholine and norepinephrine release caused by high-frequency electrical stimulation and decreasing urethral resistance and bladder outlet obstruction [84]. In another study, a significant reduction in pelvic floor pressure after onabotulinumtoxinA injection was reported in which relaxation of the pelvic floor also decreased bladder outlet resistance, especially in female patients [83]. Further laboratory evidence is necessary to clarify the mechanism of BoNT-A action in DV.

BPO is a term used to describe bladder outlet obstruction secondary to hyperplasia of the prostate (adenoma). It is the most common cause of bladder outlet obstruction in elderly men [85]. Traditionally, the growth of the prostate gland was believed to be associated with testosterone, but recently inflammation, infection, and metabolic disorders are considered possible etiologies [86]. Several clinical trials suggested transurethral intraprostatic onabotulinumtoxinA injection may be an effective treatment for patients with symptomatic BPO [87,88,89]. The prostate gland is innervated by sympathetic and parasympathetic nerves, and the parasympathetic nerves interact with the sympathetic nerves and influence secretions and contraction of the prostate [90]. Cholinergic nerves and muscarinic receptors are expressed in the prostate fibromuscular stroma, playing a role in the cell growth of the prostate tissue [91]. Therefore, blocking acetylcholine release from cholinergic nerves by BoNT-A may disrupt the neural control of the prostate, inhibit prostate contraction and growth, and bring symptomatic relief in men with BPO [92]. In a study of onabotulinumtoxinA injections in dog prostates, atrophy and increased apoptosis were observed on hematoxylin and eosin and TUNEL stains at one and three months after treatment [93]. The prostate weight, tyrosine hydroxylase-positive sympathetic nerve fibers, and synaptophysin-positive cells in the epithelium were decreased in rat prostates after onabotulinumtoxinA injection [94,95]. Although pilot studies of onabotulinumtoxinA treatment on BPO seem promising, the studies did not consistently show the same results [96,97]. A high placebo effect was noted in a double-blind, placebo-controlled phase II trial that could have contributed to the improvement of symptom scores after treatment [98]. Because the high placebo effect, BoNT-A seems not a good indication for treatment of BPO.

## 5. Conclusions

The mechanism of BoNT-A action involves the motor nervous system including the inhibition of neuromuscular junctions by blocking acetylcholine release. Desensitization of sensory nerves by reducing neurotransmitter release of ATP, substance P, and CGRP also plays a significant role. Laboratory evidence also proved BoNT-A injection regulates abnormal neurogenic inflammation and apoptosis in the bladder. Understanding of pathogenesis of LUTDs and the mechanisms of action of BoNT-A injection on these LUTDs helps clinicians properly select patients to receive BoNT-A treatment. However, not all LUTDs refractory to conventional therapy, such as BPH, IC/BPS, DV, and DSD, can benefit from BoNT-A treatment, possibly because the underlying pathophysiology of these LUTDs has not been completely elucidated. The only LUTD that can be effectively treated by BoNT-A injection is urinary incontinence due to IDO and NDO.

## Figures and Tables

**Table 1:** Mechanism of botulinum toxin A action on detrusor overactivity.

Nerve System of Botulinum Toxin A Action	Evidence of BoNT-A Effects in the Bladder	
	Changes of Neurotransmitters	Changes of Receptors
**Efferent nerve system**	Decreased Ach and ATP release in efferent nerve endings	Decreased M2 receptor in the detrusor muscle
**Sensory nerve system**	Decrease ATP and increased NO release from urothelium	Decreased P2X2, P2X3, and TRPV1 receptor in the urothelium

Ach: acetylcholine; ATP: adenosine triphosphate; NO: nitric oxide; TRPV1: transient receptor potential cation channel subfamily V member 1.

**Table 2:** Mechanism of botulinum toxin A action on interstitial cystitis/bladder pain syndrome.

Mechanisms of Botulinum Toxin A Action	Evidence of BoNT-A Effects in the Bladder	
	Changes of Neurotransmitters	Changes of Receptors
**Sensory nerve system**	Decrease CGRP, ATP, and substance P release from urothelium	Decreased P2X3 and TRPV1 receptor in urothelium
**Central nerve system**	Inhibit glutamate, dopamine, ATP, gamma-aminobutyric acid	
**Anti-inflammatory effect**	Decreased active mast cell	
	Decreased substance P, NGF, and VEGF	
**Improve urothelium dysfunction**	Decreased apoptosis in the urothelium	

CGRP: calcitonin gene-related peptide; ATP: adenosine triphosphate; TRPV1: transient receptor potential cation channel subfamily V member 1; NGF: nerve growth factor; VEGF: vascular endothelial growth factor.
